# Adaptation and Transfer of Robot Motion Policies for Close Proximity Human-Robot Interaction

**DOI:** 10.3389/frobt.2019.00069

**Published:** 2019-07-31

**Authors:** Khoi Hoang Dinh, Ozgur S. Oguz, Mariam Elsayed, Dirk Wollherr

**Affiliations:** Chair of Automatic Control Engineering, Department of Electrical and Computer Engineering, Technical University of Munich, Munich, Germany

**Keywords:** human robot interaction, motion generation, black-box optimization, dynamic motion primitives, policy improvement, close proximity

## Abstract

In the context of human-robot collaboration in close proximity, safety and comfort are the two important aspects to achieve joint tasks efficiently. For safety, the robot must be able to avoid dynamic obstacles such as a human arm with high reliability. For comfort, the trajectories and avoidance behavior of the robot need to be predictable to the humans. Moreover, these two aspects might be different from person to person or from one task to another. This work presents a framework to generate predictable motions with dynamic obstacle avoidance for the robot interacting with the human by using policy improvement method. The trajectories are generated using Dynamic Motion Primitives with an additional potential field term that penalizes trajectories that may lead to collisions with obstacles. Furthermore, human movements are predicted using a data-driven approach for proactive avoidance. A cost function is defined which measures different aspects that affect the comfort and predictability of human co-workers (e.g., human response time, joint jerk). This cost function is then minimized during human-robot interaction by the means of policy improvement through black-box optimization to generate robot trajectories that adapt to human preferences and avoid obstacles. User studies are performed to evaluate the trust and comfort of human co-workers when working with the robot. In addition, the studies are also extended to various scenarios and different users to analyze the task transferability. This improves the learning performance when switching to a new task or the robot has to adapt to a different co-worker.

## 1. Introduction

Nowadays, robots are no longer only industrial machines behind fences. Instead, they are being integrated more in our daily lives as well as in collaborative manufacturing scenarios. The new generation of robots is expected to assist elderly people in daily tasks, to support customers in markets, to work as a partner with humans in factories, etc. For all of these tasks, the robots are required to interact with the human. Especially in collaborative scenarios, where robots work with humans as co-partners in joint tasks, they need to interact more efficiently since it will increase the overall performance. Looking at the case when two humans perform a joint task as an example, the humans can anticipate each others' movements and perform a complementary action without the need of verbal communication. This facilitates teamwork and increases the efficiency of joint tasks (Erlhagen et al., 2007). Similarly, robots are expected to move in a natural way, similar to human-human interaction.To achieve such an interaction between humans and robots, the first requirement is the robot's motion must be readable to the human (Kirsch et al., 2010), which means the human partner is able to understand its intentions and the motion/behavior of the robot has to meet the expectations of the human partner. In the work of Lichtenthäler and Kirsch (2016), this is defined as legible robot behavior. Another requirement is that the robot has to be aware of its surroundings to provide a safe environment, while still being efficient in performing its task. Legibility and safety are therefore the two important criteria that increase the efficiency of joint collaboration between human and robot.

In order for humans to feel comfortable working with robots, especially in close proximity, they have to understand the robot's behavior and be able to infer their actions or in other words, the robot's behavior must be legible to the human partner. Identifying the factors that contribute to these natural movements is not trivial. According to a study conducted by Dautenhahn et al. (2005), participants want robots assisting at home to be predictable, controllable and have human-like communication. Another study (Koay et al., 2007) that investigated the subjective effects of direction of approach and distance of robots when handing an object over to humans, came to the conclusion that the frontal approach is subjectively preferred most by the participants since it is the most predictable. In addition, Bortot et al. (2013) discovered that understanding and predicting the behavior of the robot increases the well-being of humans.

The question arises how such legible robot motion can be generated. Dragan and Srinivasa (2013) tried to find one mathematical metric for legibility. However, this is insufficient as robot motion gets perceived differently by individual humans and depends on several factors including the configuration of tasks, robot positions and human positions. It is therefore necessary to have a framework in which the robot is able to learn legible motions by interacting directly with the human. In this way, all possible influencing factors will indirectly be included.

In addition, to fulfill the requirements mentioned above, legibility alone is not sufficient. In order to ensure the safety of humans in close proximity scenarios and allow joint collaborations, the robot has to know the position of the human and possibly predict their motion (Oguz et al., 2017) to modify its trajectories in real-time and reliably avoid collision with the human. Combining this safe behavior with legibility increases human comfort.

It is also worth mentioning that the main drawback of many learning approaches is the training time. The learning process usually requires several iterations of training and is time consuming to repeat for each new task and each human partner. In a lot of scenarios, it might be useful to have a flexible algorithm that still works if any parameter changes i.e., robot position, task configuration, human perspective, etc without the need of retraining. Therefore, the algorithm must be capable of extending to different scenarios and different tasks.

In this work, we develop a framework to generate legible robot motion that is transferable to different tasks and that is safe to allow collaboration in close proximity through a reinforcement learning approach. The interdependency between legibility, safety and efficiency is tackled for achieving natural human-robot interaction. Both human and robot collaborate in a joint scenario, i.e., in our case they have to reach similar objects, and the robot will adapt its motions over time corresponding to the reaction/prediction of the human partner. After training, the robot will be able to perform its tasks more efficiently and more predictable. This helps increase human comfort and the effectiveness of the collaboration. Our framework is also generalizable to similar tasks using learned policies in order to save training time.

## 2. Related Work

Safety and legibility of robot motion in close proximity have always been investigated independently. Several methods were proposed that produce real-time obstacle avoiding trajectories, while others developed optimization based algorithms for legible robot motions.

Legible (or predictable) robot motion was first introduced in Dautenhahn et al. (2005). The result from their survey confirms the necessity of predictable behaviors in future robot companions. However, the paper does not focus on how to generate predictable behaviors for the robot. In the works from the Robotics and Artificial Intelligence Group at LAAS/CNRS (Alami et al., 2005; Sisbot et al., 2007, 2008; Sisbot et al., 2010; Sisbot and Alami, 2012), they developed a human aware motion and manipulation framework which is able to generate safe, comfortable and socially acceptable motions. The framework is verified on a mobile robot manipulator in simulated environment and in a hand-over scenario on real setup. The safety criterion introduced in their works, however, is based on the distance between the robot and the human, i.e., the robot should keep its distance from the human when performing tasks. While the framework is able to generate safe and legible motion, it is not applicable for joint tasks in close proximity since it does not allow the interaction between human and robot. As shown in the results of their papers, only the robot performs its tasks and there is no collaboration between them.

The work from Dragan et al. (2013) focuses explicitly on generating predictable and legible robot motion. In their work, the authors differentiate between legibility and predictability and provide a mathematical model to produce and evaluate such motions. They assume that humans expect robots to be efficient in their movements and compare all possible goals in the scene to determine the most probable one. This probability is formulated mathematically and is being maximized for the targeted goal. This approach has some limitations. The algorithm was tested only with two goals for the robot, which the human had to predict when pausing a video which showed the robot moving to one of the two (see Supplementary Video). This setup was very simple as the probability of selecting a goal (randomly) is already 50%. Another limitation is that the subjective evaluation of robot efficiency differs from one individual to another and the algorithm does not allow to adjust the robot's movements to individual preferences of each participant.

In the work of Stulp et al. (2015), the team generates robot motions that learn from the observation of a human participant and iteratively reduce the human's reaction time. Here, Dynamic Motion Primitives (DMPs) are used for motion planning. Policy Improvement through Black Box Optimization (PIBBO) (Stulp and Sigaud, 2012) is applied to improve the robot's legibility to the human iteratively. This is done by only optimizing human guessing time about the action of the robot and the correctness of the prediction without defining formal criteria about legibility. This approach provides flexibility in choosing the relevant parameters to be optimized to obtain legible motion. Recently, Busch et al. (2017) showed that transferring the learned policy to other individuals leads to better prediction in the beginning and can thus lead to shorter adaptation times for new subjects. However, in this work no close interaction scenarios were considered as no necessary collision avoidance methods were integrated and only the policy transfer to other subjects was investigated, not the policy transfer to new tasks.

Safety for humans during interaction with the robot, in general, involves several aspects and criteria (Robla-Gómez et al., 2017). There are also different categories of methods to ensure safety for the human partner (Lasota et al., 2017) i.e., safety through control, motion planning, consideration of psychological factors, etc. Within this work, we limit the safety aspect to the obstacle avoidance behavior of the robot and therefore only mention about methods that are able to provide this functionality to the robot. In this aspect, potential field (Khatib, 1985) is a very popular and widely used approach due to its simplicity and real-time capability. Flacco et al. (2012) and Dinh et al. (2015) utilize the potential field idea in their works to provide obstacle avoidance behavior on the end-effector of an articulated robot. In the work of Park et al. (2008), the authors introduce the dynamic potential field to adapt robot trajectories while avoiding obstacles in mid-motion. This dynamic potential field is used with the inverse kinematics with null-space constraints to further ensure collision avoidance between the human and robot's links. However, the aim of these approaches was not to enable the robot to interact with humans, but rather to perform desired movements in the presence of obstacles. In a recent study by Oguz et al. (2017), a stochastic motion planning algorithm is introduced that predicts human motions and adjusts the robot's trajectories on-line to avoid the predicted region. For the prediction of the human movement, Probabilistic Movement Primitives (ProMPs) were used, which were first introduced by Paraschos et al. (2013). This method learns the distribution of the motion during training and allows prediction of human motion in the online phase. This allows close interaction between humans and robots, but does not examine predictable or legible motion.

Inspired by the work of Stulp et al. (2015) and considering the requirements of joint human-robot collaboration in close proximity, in this paper we extend the learning approach in Stulp et al. (2015) with the potential field method. Our contribution is therefore a learning framework incorporating real-time obstacle avoidance to allow humans and robots working together in close proximity and therefore both legibility and safety aspects are tackled within our framework. This means that the human partner no longer stays outside of the robot workspace as a silent observer, but really cooperates with the robot in joint tasks in the same workspace. Apart from that, we also develop a task generalization method to generate policies for new tasks from previously learned tasks. With our task generalization approach, the robot is able to adapt to new tasks faster and hence the training time is reduced. We evaluate our approach on an articulated KUKA IIWA robot in virtual reality (VR) as well as in a real robot and complete the evaluation with a human study.

In the following, we first introduce our legible motion framework in section 3 then present our idea on the task generalization method in section 4. The improvement of our framework and task generalization approach is evaluated through experiments in section 5. Sections 6 and 7 provides further discussion and concludes our work.

## 3. Legible Motion Framework in Human Robot Interaction in Close Proximity

**Figure 1:**
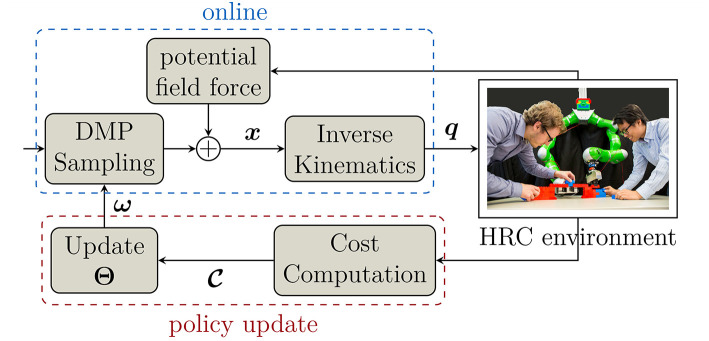
Overview of the human-guided policy improvement framework. In each iteration, DMPs generate Cartesian trajectories ***x***, which are converted to joint angles ***q*** using inverse kinematics. During interaction, potential field force is added to modify the trajectories online to provide safety when the human gets close to the robot. After execution, a cost function C is evaluated to update the policy **Θ** of the DMPs using PIBBO for the next iteration.

A general overview of our framework is shown in Figure 1. The goal of the framework is to generate legible motion for the robot directly through interaction between the human and robot. Both of them collaborate in a joint scenario, i.e., in our case they have to reach similar objects, and the robot will adapt its motions over time corresponding to the reaction/prediction of the human partner. After training, the robot will be able to perform its tasks more efficiently and more predictable. This helps increase human comfort and the effectiveness of the collaboration. The framework therefore can be described in three steps as follow:

1. Firstly, Dynamic Movement Primitives (DMPs) are used to generate smooth trajectories with modifiable parameters. These trajectories are generated in Cartesian space and converted into the joint space of the robot using inverse kinematics.
2. DMP trajectories are then executed by the robot in the online phase where the robot collaborates with the human in a joint task. During execution, a potential field force is added to modify the DMP trajectories to ensure safety of the human.
3. A cost function which evaluates how the human partner perceives each trajectory is computed. These costs are then used to update the policy, which comprises the parameters of the DMPs in our framework. In the next iteration, new trajectories are sampled based on the updated policy and the procedure repeats until it converges to an optimal predictable trajectory or the maximum number of iterations is reached.

DMP trajectories are the trials/samples that the robot performs to understand how his human partner perceives a legible motion. By changing the parameters of the DMPs, the robot is able to exploit the working area and approach the goal from different angles. The human reacts to the robot by moving to his corresponding task. Each trajectory performed by the robot is then evaluated based on the human reaction formulated in a predefined cost function. This cost function reflects the perception of the human on how legible this trajectory is. Base on the evaluation of the cost function of each trajectory, the DMP parameters will be modified in favor of the ones that are more predictable to the human (smaller costs). This is done by the policy update method called Policy Improvement through Black Box Optimization (PIBBO). After the DMP parameters (policies) are updated, the robot rolls out new samples from these parameters for the next iteration. The procedure is then repeated until the trajectories converge or the maximum number of iterations is reached. Note that all of these computations are done at the beginning of each iteration.

To prevent collision between human and robot during execution (online phase), the DMP trajectories are modified using a potential field force. This potential field force is proportional to the relative distance between the human and robot and returns an error vector that is added into the current DMP trajectory. As a result, the robot will move away when the human comes close, and recovers his task when the area is free. Additionally, in order to increase safety in close proximity, human motion is predicted using Probabilistic Movement Primitives (ProMPs) (Paraschos et al., 2013) and serves as Supplementary Information added into the potential field force. ProMPs is a recent approach that is able to generate/represent movement from a given trajectory distribution. After training with a set of human motion observations, we used ProMPs in the online phase to predict the movement of the human hand and incorporate this information into the potential field. This helps the robot react faster and can avoid the human more actively.

In section 3.1, we first briefly introduce DMPs and describe how they are used to generate smooth trajectories. The policy update method PIBBO is introduced and explained in section 3.2. This is followed by the explanation of how safety for the human partner is ensured through potential field force with the assistance of ProMPs in section 3.3. Finally, the cost function that evaluates the performance of each trajectory especially with a focus on collaboration effectiveness, is explained in detail in section 3.4.

### 3.1. Dynamic Movement Primitives

DMPs provide a method for trajectory control and planning that is able to represent complex motions and is flexible to be adjusted without manual parameter tuning or having to worry about instability (Ijspeert et al., 2002). DMPs comprise two parts, a dynamical system, and a nonlinear forcing term. In our work, the dynamical system is defined as a closed loop spring-damper system

(1) $$\begin{array}{l} \tau \ddot{y}=\alpha (\beta (y_{g}-y)-\dot{y}) \end{array}$$

that converges to the defined attractor state *y*_*g*_ where τ is the time constant, α and β are positive constants. By setting β to α/4 we get a critically damped system. The variables *y*, ẏ and ÿ are the position, velocity and acceleration, respectively.

The forcing term, which forms the second part of the DMPs, deforms the trajectory to match a desired shape. Thus, the spring-damper system is modulated to

(2) $$\begin{array}{l} \tau \ddot{y}=\alpha (\beta (y_{g}-y)-\dot{y})+f(x), \end{array}$$

where *f*(*x*) is the forcing term consisting of a weighted sum of Gaussian basis functions multiplied by a canonical dynamical system, denoted as *x*. The canonical system *x* is obtained by

(3) $$\begin{array}{l} \dot{x}=-\alpha_{x}x, \end{array}$$

where α_*x*_ is a constant. The canonical system state *x* in (3) starts at some arbitrary value and goes to 0 as time goes to infinity. This ensures convergence to the goal while keeping the forcing term not directly dependent on time. The forcing function *f*(*x*) hence has the form

(4) $$\begin{array}{l} f(x)=\frac{\sum_{i=1}^{N}\psi_{i}(x)\omega_{i}}{\sum_{i=1}^{N}\psi_{i}(x)}x, \end{array}$$

where

(5) $$\begin{array}{l} \psi_{i}(x)=\exp\left(-\frac{1}{2\sigma_{i}^{2}}{\left(x-c_{i}\right)}^{2}\right) \end{array}$$

defines the Gaussian basis functions with means *c*_*i*_ and variances σ_*i*_. In (4), *N* is the number of basis functions and ω_*i*_ are modifiable weights, which are adjusted to match the desired trajectory. They are optimized by the policy improvement method explained in section 3.2.

Since the mass spring-damper system leads to high initial accelerations, which is not desirable for robots, we use a goal system, which moves the attractor state of the system from the initial state *y*_0_ to the goal state *y*_*g*_ during the movement. This delayed goal attractor *y*_*gd*_ itself is given as an exponential dynamical system that starts at *y*_0_ and converges to *y*_*g*_.

(6) $$\begin{array}{l} {\dot{y}}_{gd}=-\alpha_{g}(y_{g}-y_{gd}) \end{array}$$

Thus the equation for the DMPs resolves to

(7) $$\begin{array}{l} \tau \ddot{y}=\alpha (\beta (y_{gd}-y)-\dot{y})+f(x) \end{array}$$

The DMPs has several advantages, which make it suitable for our framework:

- It is guaranteed to converge to the goal, since the canonical system is 0 at the end of every movement.
- The weights ω_*i*_ can be adapted to generate any desired trajectory. In our case this is especially relevant, since we want to learn the optimal trajectory and adjust the weights online with each interaction.
- As there is no time-dependency, the duration of the movement can simply be altered by adjusting τ.

### 3.2. Policy Improvement Through Black-Box Optimization

Policy improvement methods seek to optimize the parameters of a policy w.r.t. a utility function. In our work, we use a policy improvement method to iteratively update the weights of the DMP to obtain a desired trajectory. Policy improvement methods have two basic steps:

1. Exploration by perturbation: The exploration noise ***ϵ*****_*t*_** can be either added to the actions, i.e., the output of the policy (π_***θ***_(*x*) + ***ϵ*****_*t*_**), or directly to the input parameters of the policy (π_***θ***+ ***ϵ***_*t*__(*x*)).
2. Policy update: Here, the parameters of the policy are updated in order to minimize a predefined cost metric *C*. Usually, gradient descent is applied to iteratively converge to a local minimum. Another method is the reward-weighted averaging, which is used in our application.

Reward-weighted averaging does not require differentiability of the cost function, which makes it more stable than gradient descent if the cost function is not continuous.

Specifically for this work, we choose Policy Improvement through Black-box Optimization (PIBBO) as our policy improvement method (Stulp and Sigaud, 2012). PIBBO treats the whole control trajectory as a black-box, i.e., no assumptions are made about the search space or the cost function. An important property of PIBBO is that the search is done in the space of policy parameters, thus it is a parameter perturbing approach. The output *u*_*t*_ of the policy is computed as:

(8) $$\begin{array}{l} u_{k}=\pi_{\theta +\epsilon_{k}}(x),\text{ with }\epsilon_{t}\sim N(0,\Sigma ) \end{array}$$

In our case the policy π_***θ***_, is the DMP and ***θ*** are the corresponding weights for the Gaussians.

The parameter update is done using reward-weighted averaging. First, the cost *C*_*k*_ for each trajectory roll-out is computed. Then we assign higher probabilities *P*_*k*_ to trajectories with a lower cost and vice versa.

(9) $$\begin{array}{l} P_{k}=\frac{e^{-1/\lambda C_{k}}}{\sum_{k=1}^{K}e^{-1/\lambda C_{k}}} \end{array}$$

*k* is the number of roll-outs and λ is a constant between 0 and 1.

The parameter update is then given as

(10) $$\begin{array}{l} \delta \theta =\sum_{k=1}^{K}P_{k}\epsilon_{k} \end{array}$$

(11) $$\begin{array}{l} \theta \leftarrow \theta +\delta \theta . \end{array}$$

After taking the weighted average of all roll-outs, the new DMP with updated parameters ***θ*** follows the trend of trajectories with high probabilities (i.e., low costs). This process of perturbing and updating is repeated until the desired cost value is achieved or the maximum number of updates is reached.

The exploration is done by rolling out different trajectories and evaluating them using the cost values resulting from the interaction with the human. Before outlining the cost function in detail, we discuss the safety aspect of the human partner in close proximity.

### 3.3. Safety Aspect in Close Proximity

As the human works together with the robot in close proximity, safety of the human needs to be considered. In essence, the robot should be able to physically avoid the human to prevent any collision. In this section, we describe our approach to provide a safety aspect for the robot. The main idea is to create an artificial repulsive force to push the robot away whenever the human comes close (Khatib, 1990; Park et al., 2008). Furthermore, to improve the robot reactivity, the human motion is also considered. In our approach, we use Probabilistic Movement Primitives (ProMPs) to predict the human motion and incorporate its effect into the repulsive force. Our idea about generating repulsive force for obstacle avoidance will be introduced in section 3.3.1, after that, an introduction about ProMPs and how human motion prediction extracted from ProMPs is incorporated will be given in section 3.3.2.

#### 3.3.1. Repulsive Force With Artificial Potential Field

The robot trajectory is generated by the DMP at the beginning of every update. We want to modify this trajectory to avoid the human partner while still generating smooth motions and following the original DMP trajectory when the human is out of reach. As the DMP trajectory is already smooth based on its formulation (see section 3.1), the artificial repulsive force also has to generate a smooth transition on the robot. This is important for the human partner to feel comfortable when working with the robot. A simple solution is to make the robot behave like a virtual mass-spring-damper system regarding to external forces (Hogan, 1984)

(12) $$\begin{array}{l} F_{\text{ext}}=M\overset{¨}{e}+D\overset{˙}{e}+Ke, \end{array}$$

where $F_{\text{ext}}\in {\mathbb{R}}^{3}$ represents an external virtual force, which is excited whenever the human enters the safety area around the end-effector of the robot. This virtual mass-spring-damper system results in a smooth transition in the vector ***e*** ∈ ℝ^3^ regardless ***F***_ext_. This vector indicates the modification length and direction to be added to the DMP. ***M**, **D**, **K*** ∈ ℝ^3 × 3^ are positive definite matrices that represent the mass, damping and stiffness of the virtual system. In our proposed setup, ***M*** is chosen as the identity matrix, ***K*** and ***D*** are diagonal matrices chosen to adapt the desired reaction to virtual forces. Increasing the damping results in a slower reaction but smoother movement of the robot. The external virtual force ***F***_ext_ is computed based on potential fields w.r.t the distance between the end-effector of the robot and obstacles.

The idea of potential fields was first introduced in the work of Khatib (1990). Whenever an obstacle is inside a threshold region of the end-effector, a repulsive force vector ***F***_ext_ according to (12) is generated. Here, we use the same idea of repulsive vectors (Flacco et al., 2012; Dinh et al., 2015) to generate a smooth reaction force

(13) $$\begin{array}{l} F_{\text{ext}}=\frac{F_{\text{max}}}{1+\exp\left((\left\|d(E,O)\right\|(2/\rho )-1)\gamma \right)}, \end{array}$$

where **F**_max_ is the maximum force applied, ‖***d***(*E, O*)‖ is the distance between obstacle *O* and end-effector *E*, ρ is the threshold distance that defines the collision region around the end-effector and γ is a shape factor. The force reaches its maximum if the distance equals zero, and zero if the obstacle is outside the region, respectively. The steepness of the force profile within the threshold region regarding the distance can be adjusted by the shape factor γ. With ***F***_ext_, the error vector ***e*** is obtained from (12) which return in the deviation needs to be added into the DMP to avoid the obstacle.

#### 3.3.2. Human Motion Prediction With ProMP

Although the robot is able to avoid the human with the repulsive force generated from the potential field, its reaction time is an important factor that needs to be considered. In a confined workspace where the human usually interferes with the robot, the robot might not have enough time to react and fail to avoid the human partner. Increasing the safety region around the robot can improve the reaction time but results in a smaller workspace. Thus, in our framework, we estimate and predict the human motion and add this additional information into the repulsive force to increase the responsiveness of the robot.

In general, human motion estimation requires a specialized prediction method due to the inter- and intra-personal movement variations (Todorov, 2004). To imitate such behavior online, we use ProMPs and learn a distribution of a motion behavior by training with multiple trajectories performed for a specific task (Paraschos et al., 2013). ProMPs represent a discrete trajectory *X* = {*x*_*n*_}, *n* = 0…*N* defined by states *x*_*n*_ over time *N* with the formulation

(14) $$\begin{array}{l} y_{n}={\left[x_{n},{\dot{x}}_{n}\right]}^{⊤}=\Phi_{n}^{⊤}\omega +\epsilon_{y}, \end{array}$$

where ***ω*** ∈ ℝ^*k*×2^ is the weighting matrix over the *k* × 2 dimensional time-dependent basis matrix $\Phi_{n}=[\phi_{n},{\dot{\phi }}_{n}]$ with *k* being the number of basis functions and $\epsilon_{y}\sim N(0,\Sigma_{y})$ is zero-mean independent Gaussian noise, while $\Phi_{n}^{⊤}\omega$ gives the mean of the trajectory. Introducing a Gaussian distribution to also represent variance $p(\omega ;\theta )=N(\omega |\mu_{\omega },\Sigma_{\omega })$ over the weighting vector ***ω*** results in the following distribution for the trajectory:

(15) $$\begin{array}{l} \begin{array}{l} p(y_{n};\theta )=\int N(y_{n}|\Phi_{n}^{⊤}\mu_{\omega },\Sigma_{y})N(\omega |\mu_{\omega },\Sigma_{\omega })d\omega \\ \;=N(y_{n}|\Phi_{n}^{⊤}\mu_{\omega },\Phi_{n}^{⊤}\Sigma_{\omega }\Phi_{n}+\Sigma_{y}). \end{array} \end{array}$$

Using a set of motion observations, the parameters ***μ****_ω_*, **Σ**_*ω*_ can be computed by maximum likelihood estimation (Lazaric and Ghavamzadeh, 2010).

By this formulation, an online human motion prediction, where a trajectory along with the variance for each discretized time point is generated. This predicted trajectory can be used in different ways within our framework. An intuitive way is to select some predictions at different time points along the trajectory. These predictions represent the points in space where the human *might* occlude in the future and thus are treated as *incoming* obstacles that the robot has to avoid. This triggers the reaction of the robot even if the human is not currently within the safety region, which in turn increases the responsiveness of the robot. In case the human does not move toward the robot, these *incoming* obstacles do not create any disturbance, thus do not alter the robot desired position.

### 3.4. Cost Computation

In this section, we will explain how the cost function in our framework (**Figure 3**) is defined. There are different aspects that we want to evaluate through the cost function:

- First is the legibility of the robot trajectories. There are different methods to measure this aspect. In the works of Dehais et al. (2011) and Lichtenthäler et al. (2011), they show the participants robot motions and afterwards ask them to rate how legible the motions were perceived. In a quantitative level, Dragan and Srinivasa (2013) and Busch et al. (2017) show the participants robot motions through videos/experiments and ask them to indicate immediately or press a button when they feel certain about the robot's intention. Time and correctness of the prediction are used as the indicators for legibility in their works. Using the same approach as in Busch et al. (2017), we also use the human prediction time and accuracy to form the cost of legibility.
- Second is the smoothness of the trajectories. This helps the human partners feel comfortable when working with the robot and be more confident approaching their goals. Smoothness also contributes in the legibility aspect since a jerky motion does not meet the expectation of the human. In our framework, we use the third derivative of the trajectories to form the cost of smoothness.

From the two aspects that we want to evaluate, several components are identified and also mixed up depending on the experimental setup. Here, we list all the costs used in this work:

- End-effector jerk *V*_ej_: the sum of the third derivative of the end-effector position of the robot at each sample along trajectory.
- Angular jerk *V*_θ_: the sum of the third derivative of the angular positions of the controlled joints of the robot at each sample along trajectory.
- Human prediction time *V*_pred_: the time taken by the human to make a prediction about the robot's target. It starts when the robot starts moving and ends when the human reaches one of the targets.
- Accuracy *V*_task_: whether the human prediction was correct, translating to 0 cost (*V*_task_ = 0), or if the prediction was wrong which results to a cost of 1 (*V*_task_ = 1).
- Human duration *V*_dur_: the duration of the human movement between when the human starts moving and reaches the goal. It is a measurement of human's confidence in the robot's presence.
- The weighted distance between the robot trajectories, *V*_δ_, which measures how distinct the trajectory to the targeted goal is in comparison to the trajectories to the other goals. This cost is calculated using the following equation:
  (16) $$\begin{array}{l} V_{\delta }={\left(\sum_{g=1}^{G}\sum_{t=0}^{T}\frac{1}{t}\;\|p_{t},q_{t}\|{\;}_{2}\right)}^{-1} \end{array}$$
  where *G* is the number of the goals excluding the targeted goal, *g* is the other goal whose trajectory is compared to the targeted goal trajectory, *t* is the time step at which we calculate the distance, *T* is the total time of the trajectory, ***p***_*t*_ is the point at *t* in the trajectory to the targeted goal, ***q***_*t*_ is the position at *t* in the trajectory to the goal *g* and ‖***p***_*t*_, ***q***_*t*_‖_2_ is the Euclidean distance between ***p***_*t*_ and ***q***_*t*_.

In summary, the cost function has the form

(17) $$\begin{array}{l} V=\lambda_{\text{ej}}V_{\text{ej}}+\lambda_{\theta }V_{\theta }+\lambda_{\text{pred}}V_{\text{pred}}+\lambda_{\text{task}}V_{\text{task}}+\lambda_{\text{dur}}V_{\text{dur}}+\lambda_{\delta }V_{\delta } \end{array}$$

where each cost component is weighted differently. In general, λ_pred_, λ_task_ > λ_ej_, λ_θ_, λ_dur_, λ_δ_ as we want to have a high reward for trajectories that are more predictable to the human partner.

## 4. Task Generalization

Even though our framework generates predictable policies, the learning procedure requires a considerable amount of data and thus time until a convergent behavior is achieved. Furthermore, the trained polices directly depend on the specific setup. When the environment changes, e.g., the start/goal positions of the robot or the relative position of the human w.r.t. the robotic partner, the robot needs to adapt to this new configuration.

Given a fixed number of policies that have been learned on specific settings, the existing knowledge can be exploited, such that the adjustment to variations of similar tasks can be achieved given limited data. In other words, since the prior policies learned already encode some preference of human perception, they can be used to improve the learning convergence rate for the cases that the robot has not been trained for. We propose an approach to realize such a generalization capability for the policy improvement framework within HRI settings.

Suppose that the set of tasks for the robot is defined as

(18) $$\begin{array}{l} \Phi =\left\{g_{1},g_{2},\cdot \cdot \cdot ,g_{M}|M\in \mathbb{N}\right\}, \end{array}$$

where *M* is the number of available tasks. Within the scope of this work, a task is defined as a reaching motion, where the starting position is the same for all of the tasks and ***g***_1_, ***g***_2_, ··· , ***g***_*M*_ are *M* different goal positions. Learning via PIBBO is done by selecting a subset $T_{i}$ out of **Φ** and training trajectories for each goal in $T_{i}$, where

(19) $$\begin{array}{l} T_{i}=\left\{g_{i1},g_{i2},\cdot \cdot \cdot ,g_{iS}\right\}\subset \Phi ,S\in \mathbb{N},g_{ij}\neq g_{ik},\forall j\neq k \end{array}$$

with a predefined *S* < *M*. The result of PIBBO is *S* policies that generate predictable trajectories for each ***g***_*ij*_ over $T_{i}$. Each policy is parameterized by $\Theta_{T_{i}}^{ij}$, e.g., in our case given as the weighted basis functions of the DMP. Note that the policy of ***g***_*ij*_ depends on the remaining goals in $T_{i}$, which means a similar task will have different policies if it belongs to a different subset. We then denote the generated policy for a goal ***g***_*ij*_ from $T_{i}$ as

(20) $$\begin{array}{l} \pi_{T_{i}}(g_{ij}|g_{i\setminus j})=\pi_{T_{i}}(g_{ij})=\pi (\Theta_{T_{i}}^{ij}) \end{array}$$

where ***g***_*i*\*j*_ is an abbreviation of all tasks in $T_{i}$ except *j*. This can be interpreted as the policy that generates the most predictable motion for goal ***g***_*ij*_ given the remaining tasks in $T_{\;i}$.

Given a training set $T=\{T_{1},T_{2},\cdot \cdot \cdot ,T_{k}\}$ consisting of *k* batches of *S* elements from **Φ** each, a new $\tilde{T}\notin \;T$ is drawn from **Φ**. The objective here is to find a new policy for a goal $g_{m}\subset \;\tilde{T}$ such that the DMPs initialized using this policy improve the convergence rate of the learning procedure of ***g***_*m*_ afterwards. This requires finding a mapping

(21) $$\begin{array}{l} \pi_{\tilde{T}}(g_{m})=h\left(\pi_{T_{1}}(g_{11}),\ldots ,\pi_{T_{1}}(g_{1S}),\ldots ,\pi_{T_{k}}(g_{kS})\right) \end{array}$$

with ***h***(·) is a function of all policies obtained from the training set T. In fact, solving (21) is equivalent to finding the parameterized vector $\Theta_{\tilde{T}}^{m}$ in Equation (20) for goal ***g***_*m*_ in the new subset $\tilde{T}$.

We claim that a predictable trajectory for each goal in $T_{i}$ depends on a set of features **χ**. These features characterize the interrelation between ***g***_*ij*_ and ***g***_*i*\*j*_ in the subset $T_{i}$. They can be relative distances, angles, etc, depending on how the set of tasks **Φ** is defined. These features vary for each goal in each subset. Given a predefined set of *p* features for goal ***g***_*ij*_ in $T_{i}$, we denote the resulting feature vector for each goal as $\chi_{T_{i}}(g_{ij})\in {\mathbb{R}}^{p}$. We now want to establish a relation between $\chi_{T_{i}}(g_{ij})$ and vector $\Theta_{T_{i}}^{ij}$, which is the policy of ***g***_*ij*_ in $T_{i}$. Furthermore, the weighted basis functions of the DMP in $\Theta_{T_{i}}^{ij}$ are independent from each other, hence can be evaluated individually. Therefore, we propose an approximation to initialize each individual weight $\Theta \in \Theta_{T_{i}}^{ij}$ as follow

(22) $$\begin{array}{l} \Theta =\beta_{0}+\sum_{k=1}^{p}\beta_{k}\chi_{k}(g_{ij}), \end{array}$$

where $\chi_{k}\in \chi_{T_{i}}$ represents a single feature in the set of *p* features for goal ***g***_*ij*_. Given the trained policies from T and a predefined set of features **χ**, ***β*** = {β_0_, ***β***_*k*_} in (22) is obtained by solving the linear regression problem. Assuming $\Theta_{T_{i}}^{ij}$ has *N* basis functions, then *N* linear regression problems of (22) are solved individually to obtain *N* sets of ***β***, denoted as ***β***_*i*_ where *i* denotes the according basis function index.

From there, given the new subset $\tilde{T}$, the generalized policy for a goal ***g***_*m*_ in $\tilde{T}$ is initialized by the approximate value as

(23) $$\begin{array}{l} \Theta_{\tilde{T}}^{m}=\left[\begin{array}{c} \beta_{1}\\ \vdots \\ \beta_{N} \end{array}\right]\chi_{\tilde{T}}(g_{m}), \end{array}$$

where $\chi_{\tilde{T}}(g_{m})$ is the features of ***g***_*m*_ in $\tilde{T}$. We then use this policy as an initialization for the DMP when learning predictable motion for the new subset $\tilde{T}$. Details about our implementation and results are outlined in section 5.3.

## 5. Results

In this section, we present different experiments to evaluate our framework and task generalization method. In section 5.1, we first describe the experimental setup in virtual reality (VR). The legibility results are shown in section 5.2 while in section 5.3, we present the results of our task generalization approach. Finally, to verify the safety aspect of our approach we conducted an experiment on a real KUKA LWR 4+ robot and present its results in section 5.4.

### 5.1. Experimental Setup in Virtual Reality

**Figure 2:**
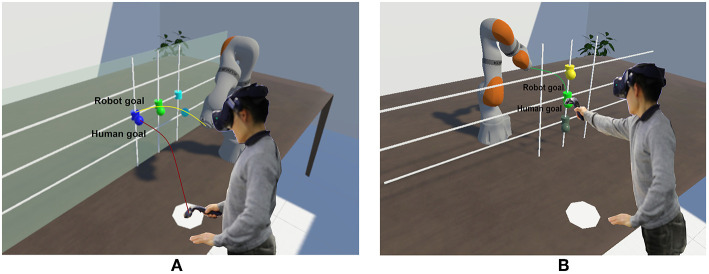
Experiment setup with different configurations: **(A)** Human and robot are on the same side. **(B)** Robot is on the opposite side of the human.

We conduct our main experiments in a VR environment as shown in Figure 2. There are advantages of VR that facilitate our work: First, it is easier to change the environment or switch to different robots and second, VR provides a first person point of view that is similar to how humans would perceive their environment, which makes it suitable for our work.

In the experiment, the participant wears a VIVE pro headset and stands in front of a table with the robot mounted on it in VR. We added a real table at the exact location as in VR, which both acts as a physical support and improves the realism of the interaction. The position of the robot is different depending on the experiments. In our case, we use two configurations: (*i*) the robot is mounted on the same side of the participant, and (*ii*) on the opposite side of the participant relative to the collaborative task area. The first case emphasizes the side-by-side perspective of the human toward the robot motions and the second case highlights the direct point of view when the human observes the robot motions from the opposite side. Here we want to investigate if this perspective also affects the predictable motion of the robot. To facilitate the collaboration between the human and the robot, we design the tasks for both as reaching designated goals. The goals of the robot are visualized as cylinders and the goals of the human are visualized as spheres. Each goal of the robot has a corresponding goal of the human with the same color. They are positioned near each other to evaluate obstacle avoidance behavior (Figure 2).

For each experiment, there are three different goals for the robot and three corresponding goals for the human. The robot starts first by moving to one randomly chosen goal and the participant has to predict which one the robot is aiming at and moves the VR controller to the corresponding goal with same color when they feel confident about the target of the robot. After that, both the participant and robot move back to their starting positions and the procedure repeats. The participant is informed that this is a collaborative task, therefore they are expected to find a balance between making a correct prediction or being fast and reacting early. For example, making many wrong predictions results in failing the tasks, whereas having long prediction time increases the total amount of time for both to finish their tasks. Both cases reduce the efficiency of the collaboration.

Each experiment consists of a habituation phase and an evaluation phase. The purpose of the habituation phase is to get the participants acquainted to the VR environment and the used equipment as well as familiarized to the robot motions and their own task. This habituation phase reduces the learning effect during the main evaluation phase. During the evaluation phase, the participants are asked to answer a questionnaire. The answers are scaled onto 5 different levels: *strongly disagree, disagree, neutral, agree, strongly agree*. There are 11 questions in total, that are classified into 5 categories:

- How does the participant feel about the smoothness of the trajectories?
- Does the participant feel safe when working with the robot?
- Are the robot trajectories predictable?
- How natural and comfortable the participant feel about the robot trajectories?
- How does the participant like and want to work with the robot again?

The user study was approved by the ethics committee of the TUM School of Medicine. All subjects gave written informed consent in accordance with the Declaration of Helsinki.

For all experiments, if not mentioned specifically, we use configurations and parameters described as follow:

- For DMP, we use three goal systems for the three Cartesian goal positions of the end-effector. These goal systems are first initialized with straight lines. The DMP has 5 equally spaced Gaussian radial basis functions and there are 5 samples per update for each goal. In the sampling phase, we add perturbations with the covariance size as 200 to the DMP parameters and run the policy for each sample. With each iteration, we let the variance factor for the perturbations decay as it helps reducing the search space for the parameters over time.
- For obstacle avoidance, we use a motion capture system to detect the position of the human (and the velocity), which are then used to compute the repulsive force. The maximum force ***F***_max_ is set to 300*N* and the obstacle threshold is 20 cm around the end-effector of the robot.
- The weights of cost components used in the experiment are: λ_ej_ = 1, λ_θ_ = 2, λ_pred_ = 8, λ_task_ = 10, λ_dur_ = 1, λ_δ_ = 3. The weights of the human prediction time and accuracy costs are relatively higher than the others.

Over time, the policy of the robot is updated to adapt to the preferences of the human and produces more predictable movements to the human partner. The results of this adaptation are presented in the following section.

In order to convert the Cartesian trajectory produced by the DMP into joint positions, we use traditional inverse differential kinematics:

(24) $$\begin{array}{l} \dot{\theta }=J^{+}\dot{x} \end{array}$$

with ***J***^+^ being the pseudo inverse of the Jacobian ***J*** of the end-effector (Penrose, 1955), ***θ*** ∈ ℝ^7^ is joint configuration and ***x*** ∈ ℝ^3^ is Cartesian position. The pseudo inverse gives the least square approximation to the real inverse. In our case only the pseudo inverse is applicable, as we map three Cartesian values to seven joint positions, which makes the Jacobian not quadratic and thus not regular. We constrain the covariance size of the DMPs to avoid generating trajectories out of the robot's reach. In addition, the joint configuration corresponding to the starting position is fixed for all trajectories. In this way, the elbow position of the robot resulting from joint redundancy does not change significantly during the experiment. Hence, the adaptation effect is mainly visible on the end-effector movement. The motion of the end-effector is formed based on the DMPs trajectories and the potential field force applied to it and it is the major factor for the human partner to differentiate between different robot motions. Our detailed implementation is provided in https://github.com/khoilsr/hrc_legible_motion_generation.

### 5.2. Predictable Robot Motion for a Specific Setup

Given a specific setup, which in our case comprises the goals of the human and the robot in addition to the robot mounting position (either in the same side or opposite side of the human), the predictable trajectories are obtained through the learning framework. We conduct experiments with different participants on different configurations to evaluate overall performance. To quantify the performance of our framework, we look at the following criteria:

- The total cost *V* and human prediction time cost *V*_pred_ (section 3.4) for each update. *V*_pred_ is used to quantify the legibility of the robot motion while *V* shows the overall efficiency of the learning framework.
- The opinion of the subject about how legible robot motions are after each phase.
- The converged trajectory for each goal after learning w.r.t each subject.

The first two criteria will be discussed in sections 5.2.1 and 5.2.2 while the last one will be analyzed in section 5.2.3.

#### 5.2.1. Evaluation of the Learning Framework

Fifteen participants took part in this study. As mentioned, each experiment consists of a habituation phase and an evaluation phase. In the habituation phase, 30 trials are executed using invariable DMP trajectories. After its completion, the evaluation phase starts, which consists of 10 updates with 5 trials per update for each of the three goals, resulting in 150 trials in total. This number is comparable to Stulp et al. (2015) and Busch et al. (2017). To evaluate the participants' perception during the experiment, this phase is divided in three blocks with two breaks after the 4^*th*^ and 7^*th*^ update, respectively, in which the participants are asked to fill a short questionnaire (see results in **Figure 4**).

The prediction time and accuracy from all participants is collected using a motion capturing system after each trial to update the cost function and evaluate the framework over time. The human prediction time is calculated by measuring the time between the start of the robot's motion until the participant reaches their goal. Since each human being has a different inherent reaction speed, we normalize the measurement of the human prediction time of each participant by their responses on the first update, which is computed as the average of 15 values of the human prediction time.

**Figure 3:**
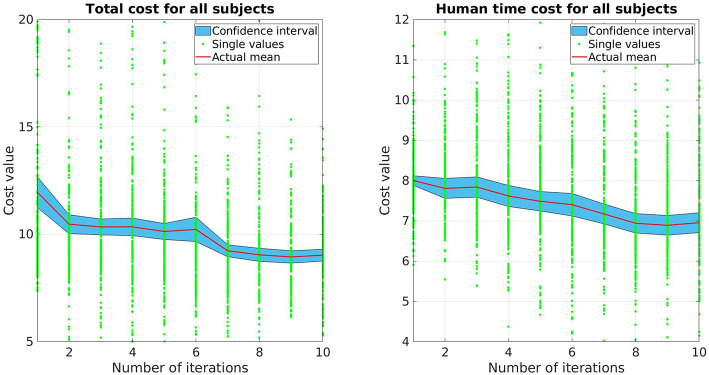
The mean and confidence interval of the total cost and human prediction time cost for all subjects.

Both total cost *V* and human prediction time cost *V*_pred_ for all subjects are presented in Figure 3. For each update, there are 225 data points (15 trials per update for 15 participants), each data point represents the measurement of one single movement of the participants. The red line depicts the mean while the blue area illustrates the 95% confidence interval. As shown, both cost values decrease over time. The human prediction time cost *V*_pred_ drops around 13%, while the total cost *V* drop is around 24%. Comparing the data between the first and the last update, a pair-sampled *t*-test indicates that they are both significantly different from each other (*t* = 7.142, *p* < 0.001 for *V* and *t* = 7.437, *p* < 0.001 for *V*_pred_). The decrease of human prediction time indicates that the subjects are able to predict and react faster to the robot motions while the reduction of total cost also implies that the subjects predict more accurately over time (accuracy cost has the highest weight).

**Figure 4:**
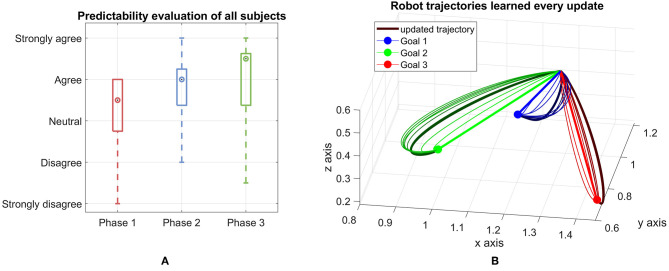
**(A)** Predictability evaluation from all subjects for each phase. The evaluation of participants for the first 4 updates, then the next 3 updates and the last 3 ones are shown in phase 1, phase 2 and phase 3, respectively. The mark shows the median of each group. The box contains 50% of the middle half of all given answers thus representing the interquartile range of the data. The whiskers mark the most extreme answers. **(B)** Robot trajectories learned for each goal every update. The first updates (straight lines) and last updates are marked thicker than the others.

The subjective legibility of robot trajectories is measured by the questionnaire during the breaks and after the last update. Here, we asked the participants' opinion on two statements: *the robot's intention was clear* and *it was easy to predict which goal the robot is targeting*. We get the average of the two answers as the measurement of legibility aspect from the human perspective. The trajectories become more predictable as the median increases over time (Figure 4A). An interesting result that can be observed here is that the interquartile range is reduced from phase 1 to phase 2, however it slightly increases from phase 2 to phase 3. This means the improvement from phase 2 to phase 3 is not very clear as the mean increase but the data spread is also larger. One reason for this is due to the trajectories of the robot start to get close to the converged one after a few updates and the updated trajectories of phase 2 and phase 3 are quite close together. An example of this behavior is shown in Figure 4B, where the trajectories start as a straight line toward the goals and after a few updates, get close to the converged trajectories depicted as the bold and dark curves for each goal.

Overall, it can be concluded that, given a specific setup, human prediction time and subjective legibility can be improved through our framework and therefore can boost the efficiency of the collaboration between human and robot. However, the question arises here whether the learning effect of the participants plays a significant role in the improvement of the results, since the experiment is designed as a repetitive task. This will be discussed further in the next section.

#### 5.2.2. Comparison With Non-adaptive Robot

In this section, we compare our method with a non-adaptive baseline. Even though we reduce the learning effect from the participants through the habituation phase, there is still probability that the human adapts to the motions of the robot over time. Therefore, the goal of this section is to investigate if the prediction of the human is improved due to the legible motions of the robot or because of human adaption. We design two experiments with the same environment setup, i.e., the tasks and the positioning of human and robot are the same. We use the counterbalanced ABBA design and define the following two groups:

- Group I: Subjects within this control group first interact with the non-adaptive robot, then with the adaptive robot subsequently.
- Group II: Subjects within this control group first interact with the adaptive robot, then with the non-adaptive robot subsequently.

In the case of non-adaptive robot, we also use our framework, but the policies (the parameters of the DMP) will not be updated. Therefore, the non-adaptive robot will always follow a straight line from the start toward the goal in every motion. As there is no adaption from the robot, the results from the non-adaptive robot solely reflect the learning capability of the human over time. This configuration also guarantees that the trajectory of the robot is smooth based on the DMP formulation (section 3.1) and the avoidance behavior is identical to the adaptive robot. The only difference between the two robots is the method to generate their motions which can be evaluated by comparing the results from the two experiments.

The experiments are then conducted on 14 new subjects, divided into 2 groups of 7 participants each. The procedure for each experiment is identical to the experiment described in section 5.2.1.

**Figure 5:**
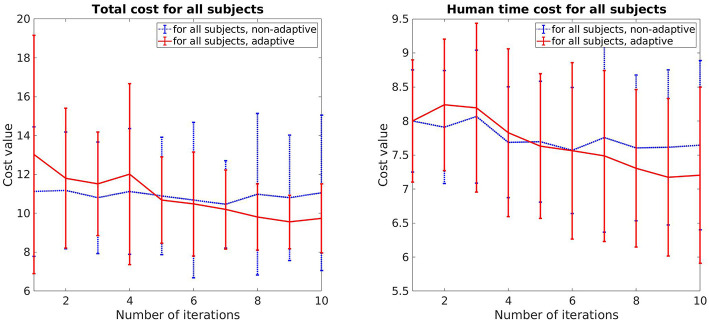
Comparison of the total cost and human prediction time between adaptive robot and non-adaptive robot.

The total cost and human prediction time for both cases, adaptive and non-adaptive robot, are shown in Figure 5. The error bar represents the mean value and standard deviation for each update. For the adaptive robot, there is a clear tendency for decreasing in both total cost and human time cost over the course of iterative updates. On average, the total cost deceases around 22% and human time cost decreases around 10%. In the case of non-adaptive robot, these values are 3.8% and 4.5%, respectively. It can also be seen that for the first few updates, the subjects collaborate better with the non-adaptive robot as both of the costs are lower. This is due to the fact that the adaptive robot uses a trial and error method to understand how the human perceives legibility by exploiting different motions. Motions that are harder to predict result in a higher cost, as shown in the slightly increasing in the human time cost on the second and third updates of the adaptive robot. But overtime, its motions become more predictable and easier for the subjects to predict compared to the non-adaptive robot, as indicated by the better performance in both cost values from the sixth update and after.

We also perform pair-sampled *t*-test to evaluate how significantly different is the performance between the adaptive and non-adaptive robot. On the first update, the performance between both robots is not significantly different (*t* = −2.464, *p* > 0.001 for the total cost *V* and *t* = −0.266, *p* > 0.001 for the human prediction time cost *V*_pred_). In contrast, on the last update, the *t*-test results in *t* = 4.139, *p* < 0.001 for *V* and *t* = 3.185, *p* < 0.001 for *V*_pred_, which indicates that the difference is significant. Overall, our conclusion drawn from this section is that the improvement in the human prediction time and the overall performance is mainly from the legible behavior of the robot. The learning effect from the human partner, while also reducing the human time and cost, does not have a significant contribution within our framework.

#### 5.2.3. Predictable Trajectory Evaluation

**Figure 6:**
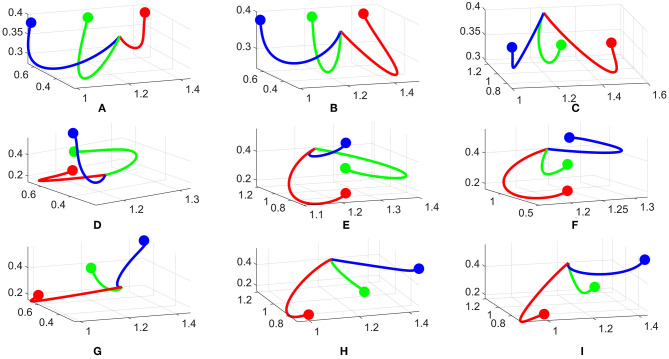
Converged trajectories from different subjects and configurations. **(A–C)** Horizontal, **(D–F)** Vertical, **(G–I)** Diagonal configurations.

**Figure 7:**
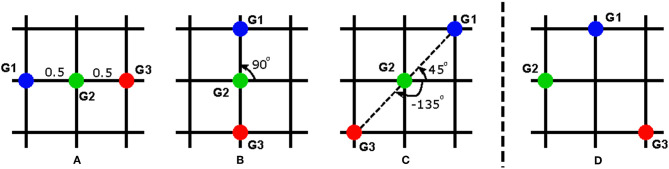
Different configurations of the robot goals: **(A–C)** are used for training, **(D)** is used for testing the task generalization approach.

To analyze the converged trajectories from the policy improvement framework, we first pick three different configurations: 3 goals in a horizontal line, 3 goals in a vertical line and 3 goals in a diagonal line. These configurations are illustrated in **Figure 7**. Combined with two different mounting positions of the robot (same or opposite to the human), we have 6 cases in total. The experiments are conducted with several participants for each case. In Figure 6 we representatively show 3 converged robot trajectories for each goal configuration.

For the horizontal configuration, Figures 6A,B are with the robot on the same side and Figure 6C is with the robot on the opposite side. The robot tends to bend more on the left or right for the blue or red goal, respectively, while for the green goal, the robot tries to keep the trajectory in the middle with a small variance, i.e., the green line diverges slightly to the left side in Figure 6A. Another variance is the length of the trajectories, e.g., the red line is the shortest in Figure 6A and longest in Figure 6B. All trajectories tend to go downward for all three results.

The vertical configuration is one of the most interesting case as the trajectories converge quite differently. For example, the green line curves to the left in Figures 6D,E but keeps in the middle-left in Figure 6F. The red line is the only one bending to the left in all three results. However, we observe the same pattern for all three results. For each case, one trajectory bends to the left side, one to the right side and one stays in the middle. This creates a divergence between the three trajectories and makes it easier to predict. The difference in trajectory shape toward each goal comes from the random sampling of DMPs during the rollout phase. For example, if there are more rollouts for the green goal to the left side and being predicted correctly by the human, these rollouts will be rewarded more and push the next update to the left. Another reason is the personal preference of each participant, i.e., for the blue goal, it is easier for one participant to predict if it bends to the left side, but for another the right side is favorable. Hence, these trajectories are rewarded differently.

For the diagonal configuration, we observe similar behaviors as in the horizontal one. The green line stays in the middle while the two others diverge to the corresponding directions. Also in this configuration, the distance between two goals is larger than previous cases, therefore it is easier for the human to predict in this configuration. The blue line is one example as it tends to go straight toward the goal in Figure 6H.

For all configurations, we observed slightly different trajectories w.r.t the mounting position of the robot. It seems the perspective affects the shape, but it's not always significant. This is probably because from the human point of view, the shape of trajectories does not change a lot, therefore it does not affect the predictability too much.

In summary, there are differences between trajectories w.r.t different subjects and configurations i.e., length, bending angle, etc. However, we also observed several similarities and patterns in the robot trajectories that make them become more predictable to the human. This motivates us to learn these patterns such that they can be generalized to other cases.

### 5.3. Task Generalization Evaluation

As learning a policy for each task and each configuration requires considerable amount of time, it is preferable to take advantage of the knowledge of the prior polices as it already encodes some preference of human perception. In this section, we evaluate our task generalization presented in section 4. To generalize the policy for task ***g***_*m*_ in a new set $\tilde{T}$, we have to find a set of features $\chi_{\tilde{T}}(g_{m})$ (see section 4). From our observation and from the results in section 5.2.3, we identified some critical features that a predictable trajectory depends on:

- The relative distances from the target goal ***g***_*m*_ to other goals in $\tilde{T}$.
- The angles between the target goal ***g***_*m*_ to other goals in $\tilde{T}$ w.r.t the horizontal line.
- The relative angle between the human and the robot.

Without loss of generality, we illustrate our idea for the case $\tilde{T}$ consisting of 3 goals as depicted in Figure 7. The workspace of the robot is divided into a 3 × 3 lattice where robot goals can be located in 9 different positions. For the sake of simplicity, the height of the workspace is normalized as 1. Figure 7 depicts some possible configurations and how $\chi_{\tilde{T}}(g_{m})$ is calculated. For example, for *G1* in Figure 7A, the relative distances to *G2* and *G3* are 0.5 and 1, respectively, the angles to *G2* and *G3* are both 0°. For *G2* in Figure 7B, the angles are 90° and -90° while for the same *G2* in Figure 7C, these values are 45° and -135°. The relative angles between the human and robot is set 0° if the robot in mounted on the same side with the human and 180° if the robot is mounted on the opposite side of the human. Within the scope of this work, we only investigate these two mounted positions of the robot, but it can be extended to other cases, e.g. the robot is positioned on one side of the table such that the perspectives of the human and robot are orthogonal.

To verify our task generalization approach, three configurations Figures 7A–C combined with two different robot positions are used for the training phase (6 different cases in total). The training phase consists of 18 subjects, equally distributed for all cases. For each experiment, we obtain the policy w.r.t each subject for each case. The weights of the converged trajectories are extracted to construct a regression model. Then, we use a new setup depicted in Figure 7D with the robot mounted on the same side with the human as a testing sample. Using the corresponding features for the new setup as the input, we initialize the DMP with the output of the regression function in Equation (23).

**Figure 8:**
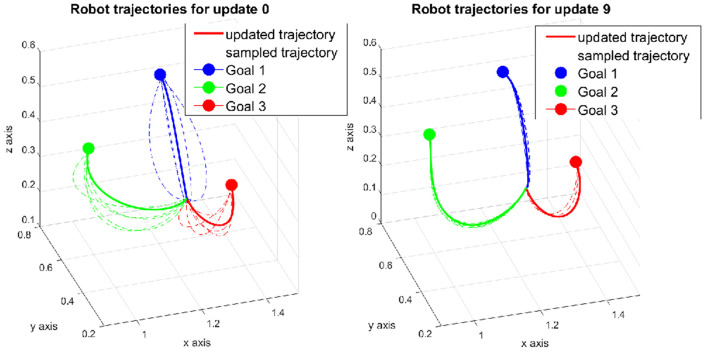
Robot trajectories in the task generalization experiment. The trajectories were initialized by the weights generated from the regression model.

The robot trajectories in the first and final update are depicted in Figure 8. The trajectories are initialized as curves toward the three goals in the first update instead of straight lines in the non-trained case. For *G1*, the curve bends upward while for *G2* and *G3*, the curves deviate downward, more to the left and right from the human point of view, respectively. These behaviors match the expectation that we observed in section 5.2.3. During the updates, the robot continues exploring new motions around the initial ones. The covariance size of the DMP perturbation is set to half of the value of the non-trained case so that the rollout trajectories are sampled in a smaller area. The converged trajectories for each goal are shown in the final update in Figure 8. Compared to the first update, the shape of the trajectories does not change a lot, which indicates that the learning algorithm stays close to the minimum from the beginning.

Next, we analyze the outcome of the total cost and the human prediction time. Our goal here is to compare the performance of the learning method to the non-trained case. Therefore, we establish two groups with 6 new participants each:

- Group A: Subjects within this control group interact with the untrained robot on a specific experimental setup different from the ones used for training the data.
- Group B: Subjects interact with the robot, whose trajectories are initialized by the regression model. The experimental setup is identical to the one of Group A.

**Figure 9:**
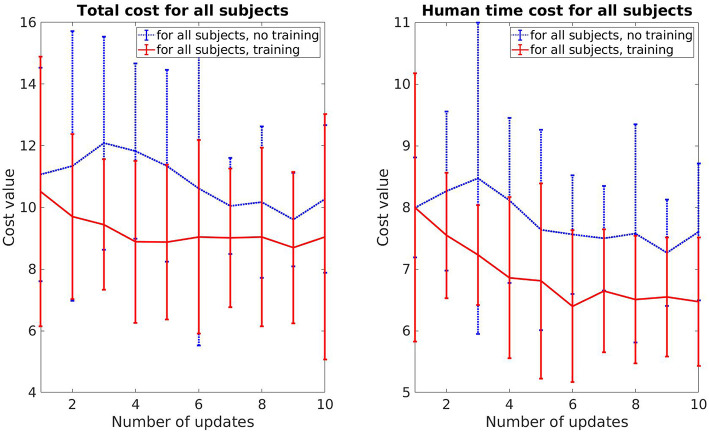
Cost plots that show the difference between the control group that interacted with the untrained robot and the results for the interaction with the trained robot.

The experiment procedure is the same as described in section 5.2.1. The human prediction time cost of each subject is also normalized for cross comparison. The means and standard deviations of the total cost and human prediction time cost from both groups are plotted together for comparison (Figure 9). A clear improvement of the trained robot can be observed directly from the result as both the total cost and the human prediction time cost are lower than the untrained robot. In addition, the cost values of the trained robot start decreasing from the start while in the case of untrained robot, they start increasing at first then decrease due to high exploration in the beginning. As the experiment is designed exactly the same between both groups, the improvement of the trained robot comes from the initialized trajectories derived from our task generation approach. Instead of exploring the whole area, the trained robot only needs to search around the given trajectories, which inherit the properties of legibility from training data. As a result, the human predicts easier and faster over time, i.e., the human time cost drops substantially 20% in the case of the trained robot compare to 10% of the untrained robot after 10 updates.

As a conclusion, the task generalization approach that we proposed increases the efficiency of the learning framework. Starting from an initial trajectory generated from the approach, the robot trajectory converges quickly to the predictable one, which is also close to the initial trajectory. This helps to reduce the number of updates and the number of sampled trajectories per update, which in turn reduces the amount of time needed for training.

### 5.4. Experimental Results on a Real Robot

**Figure 10:**
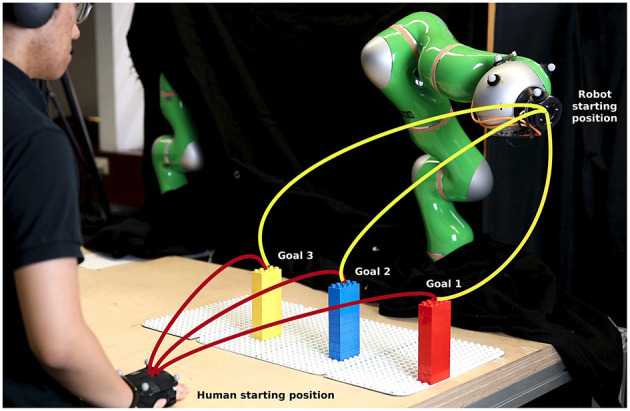
Real experiment setup on a KUKA LWR 4+ robot.

As shown in previous sections, our approach is efficient in learning predictable motions for the robot through interaction in VR. We take one step further and bring our framework into a real robot. While performing the experiments in VR allows us to evaluate our hypotheses in different setups and configurations without the need to account for the system limits, safety, etc. in the performance, it is difficult to judge the safety aspect from the human perspective since there is no real collision possibility during the experiment. Therefore, the safety aspect is additionally evaluated in this section. For this purpose, we design the experiment as illustrated in Figure 10 with the robot on the opposite side of the human. The robot used in this experiment is the KUKA LWR 4+ which has 7 degrees of freedom. The same inverse kinematics introduced in section 5.1 are applied to convert the Cartesian position to joint configuration for the robot. The trajectories generated from our framework are sent to the robot via ROS (Robot Operating System) at the frequency of 100*Hz*. The KUKA robot uses the joint position control internally to keep track of the sent trajectories. Slightly different from the setup in VR, here the goals of the human and robot are chosen to be the same and are constructed in the form of three LEGO blocks (red, blue, and yellow). With this configuration, the human needs to enter the robot workspace to reach the goals and therefore triggers the possibility of collision at every movement. The human hand and robot end effector are equipped with passive retroreflective markers which are tracked by a Qualisys tracking system. This information is then used by the robot to avoid the human and provide safety during the experiment.

The experiment procedure is then designed identically to previous sections with a habituation phase and three main blocks in the evaluation phase. The first block contains 4 updates while the second and third one contain 3 updates each. After each block, there is a short break for the subject to answer a questionnaire. The questionnaire is designed similarly to section 5.2.1 with the same questions about the legibility of robot motions. Additionally, new questions are added to evaluate the safety aspect and comfort of the participants. For safety, we asked the participants' opinions about three statements: *The robot is responsive to my movement, The robot does not hit me while moving* and *I feel safe working with the robot*. The first two statements focus on the avoidance behavior of the robot since this is the key feature to provide safety for the human. The last statement is a direct question to the participants if they feel safe when working with the robot. The average of three answers is used as the measurement for safety aspect. Similarly, for comfort, two statements were asked: *The motion of the robot is natural to me* and *I feel comfortable working with the robot*. Here we want to evaluate if our framework also provides comfort to the human partner. The experiment lasts around 30 min in total. During the experiment, the participants are asked to wear a headphone with concentration music so that they do not get distracted by the surrounding environment.

**Figure 11:**
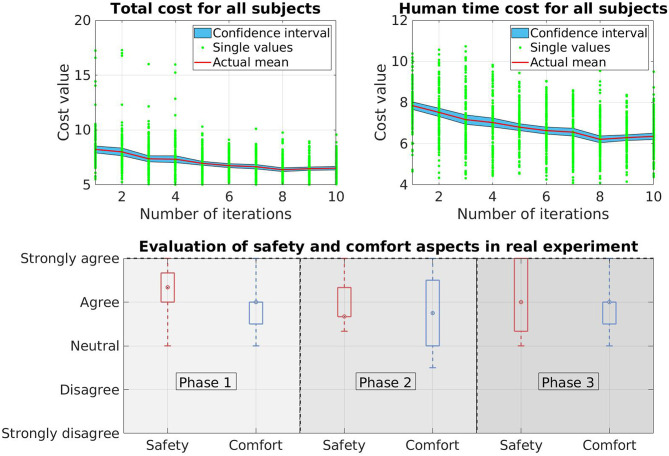
Results of the real experiment on a KUKA LWR 4+ robot. The top side of the figure shows the total cost and human prediction time cost while the bottom side shows the human evaluation in the safety and comfort aspects when working with the real robot.

We collect data from 10 new participants who have not participated in or known about the VR experiments. The results therefore only reflect the performance of the real robot. Regarding the total cost *V* and the human prediction time cost *V*_pred_, we observe similar patterns as in VR experiments. Both cost values decreases over time (Figure 11). In this case, the total cost *V* drops around 20% and for the human prediction time cost *V*_pred_, the drop is around 19%. The improvement in the cost values indicates that the trajectories of the KUKA robot is more predictable over time.

The bottom side of Figure 11 shows the evaluation of safety and comfort aspects in box-plot. In case of safety, there is almost no negative answer from the participants as the data spreads only from *neutral* to *strongly agree* in all three phases of the experiment. The boxes, which contains 50% of the answers spread around *agree* level in phase 1 and phase 2. In phase 3, there is a larger variation since the box spreads from above *neutral* to *strongly agree*. In general, the data shows positive feedback which means the participants are confident that the robot will not hit them while moving and therefore they feel safe when working with the robot. Some participants, that we observed that during the experiment, even show their interest in the behavior of the robot by repetitively interacting with the robot after finishing their task (they keep moving their hand toward the robot to see how the robot reacts to their movement). For comfort, the participants also give positive feedback as most of the answers are above *neutral* level. Only in phase 2, one of the whiskers stays below *neutral* level. However this is an extreme case (1 out of 10 subjects) which also reflects the difference in subject's personality. Overall, we can conclude that our framework is able to provide a safe and comfortable environment for the interaction between human and robot during the learning process.

## 6. Discussion

Our learning framework is a framework that combines learning and interaction into one. By ensuring safety for the human partner, we are able to change from “learning from observation” to “learning through interaction.” The results in sections 5.2.1 and 5.2.2 show that our framework is able to generate motions that are legible to the human partner during interaction. A substantial improvement compared to the non-adaptive baseline also points out that the robot motion is more legible over time due to its own adaption and the learning effect from human does not play a significant role during the learning process. We also present some preliminary results in our task generalization approach. We first learn the policies of three sampled tasks and use our approach to generate the policy for a new one. Results presented in section 5.3 indicate that the robot initialized with this policy achieves a better performance. This confirms our hypothesis that legibility can also be transferred to similar tasks and our framework therefore is generalizable using our task generalization method. We also verify our framework in a real experiment setup and show that it is able to provide a safe environment for the human partner. Even though the results that we presented show the effectiveness of our framework, there are some other aspects that we want to discuss in detail.

In our study, we evaluate and verify different hypotheses as presented in section 5. Beside that, there are also other case studies that are worth investigating in further experiments. One case study that is interesting to further investigate is how the predictable trajectories learned from the framework are affected by the relative perspective of the human and robot. The motivation of this study comes from the fact that the human partner usually does not stay at a fixed position, but rather goes around when working with the robot. Therefore the robot trajectories also change from the human point of view. In our work, two mounting positions of the robot were evaluated and we obtained some preliminary results. However, further positions need to be investigated to justify this proposition. Another case study is about the variation in perception of different types of participants, e.g., participants who have robotics background behave and react differently when working with the robot compared to others who do not have robotics background. Comparing the outcomes of the learning framework from these types of participants requires further inspection but might lead to interesting results.

Task generalization is a concept to estimate the policy for a new task from the existing policies of the prior trained tasks by exploiting the relation between human perception in term of predicting robot trajectories and task specifications. As a result, for a new task, the robot starts from a trajectory that is more predictable to the human and therefore the convergence rate of the learning framework is improved. We demonstrated our idea in a 3 × 3 lattice environment with 3 tasks for the robot per configuration and showed the effectiveness of the approach. The advantages of our method are: First, it does not require the exact positions of tasks but only the relative positions between them as we only estimate the basis functions of the DMP; second, it can be extended to an *n* × *n* case with larger number of tasks per configuration without lots of modifications. However, since the features that specify the differences of tasks are defined from the start and do not change during the learning phase, the variation of new tasks whose policies can be estimated by our approach are limited. The reason is these new tasks need to be described using the same features. For example, in our work, all tasks or the robot are reaching a goal on a vertical plane.

With the promising outcome of the task generalization method, there are some consequent open questions that are worth investigating further. The first question is how to identify features and how to qualify the influence of each feature to the trajectories of the robot. In this work, we did it mainly by observing from a certain number of participants and identifying some critical features. However, more data is required to properly justify these features. Another interesting question is how many cases are needed for the training phase of the task generalization approach and how to select these cases such that it comprises enough information about the interrelation between tasks. Too many training cases requires lots of training time, thus reduces the efficiency of the approach. But too few training cases might not contain enough variation, therefore affect the outcome of the generalization method.

Finally, the experiment on the KUKA LWR 4+ robot is our first step to bring our learning framework to reality. The avoidance behavior of the robot is reliable such that the human feels safe and confident to cooperate with the robot. Here, we want to emphasize the importance of this avoidance behavior and its contribution on the success of the learning process since it allows a smooth and consistent behavior from the human partner in term of prediction and hand movement. One example is that in case of collision during the experiment, the human would feel uncomfortable and hesitant to do the next movements, which may lead to inaccurate measurement of the human prediction time. Beside that, one limitation in our setup on the KUKA LWR 4+ is the working area of the robot. Due to the joint limits of the robot (especially the elbow), the mounting position and our configuration to avoid singularity, the workspace of the robot is quite small as we can only setup 3 goals with the distance between them being around 20 cm. As a result, it is difficult to extend the framework to different tasks and evaluate the task generalization method in a real setup. A solution for this is to change the mounting position i.e., mount the robot on the ceiling to have a larger range on the elbow or use a different robot with larger working space.

## 7. Conclusion

In this work, a framework is developed to generate predictable robot motion that can adapt to human preferences and can avoid dynamic obstacles, which in our case is the human hand during interaction. The experiments that were conducted show that robots are able to adapt their behavior to human preferences. They can learn to become more predictable while still giving humans the freedom to move safely in the same work space. The humans became faster and more confident in their predictions. Furthermore, a task generalization approach is also developed and tested. In our experiment, the learned policy produces better results in the new task than the control group without a pre-learned policy. This confirms our hypothesis that the policy learned by this framework is indeed transferable to other tasks.

## Data Availability

No datasets were generated or analyzed for this study.

## Ethics Statement

The user study was approved by the ethics committee of the TUM School of Medicine. All subjects gave written informed consent in accordance with the Declaration of Helsinki.

## Author Contributions

KH and OO conceived of the presented idea and developed the framework and formulated the formulations. ME provided inputs for the framework. KH and ME performed the experiments and collected data. OO and DW verified the approach and supervised the experiment. All authors discussed the results and contributed to the final manuscript.

### Conflict of Interest Statement

The authors declare that the research was conducted in the absence of any commercial or financial relationships that could be construed as a potential conflict of interest.
